# Eating Disorders in People Who Identify as Gender-Diverse: Associations Between Gender Diversity, Eating Disorder Diagnosis, Minority Stress Experiences and Mental Health Comorbidity

**DOI:** 10.3390/nu18030458

**Published:** 2026-01-30

**Authors:** Rebecca Murphy, Helen Sharpe

**Affiliations:** Department of Clinical and Health Psychology, School of Health in Social Science, University of Edinburgh, Edinburgh EH8 9YL, UK

**Keywords:** eating disorder, anorexia nervosa, bulimia nervosa, transgender, gender identity, gender-affirming care, mental health

## Abstract

**Objective:** Mental health (MH) comorbidity is elevated in individuals who identify as gender-diverse (GD) and in individuals with an eating disorder (ED). GD individuals with an ED may have significantly higher MH comorbidity than individuals with just one of these conditions. Gender Minority Stress Theory suggests that experience of stressful events associated with gender minority status may increase risk of developing MH difficulties and may explain the elevated risk of ED diagnosis in GD populations. **Method:** This is a cross-sectional analysis of survey data. Using data from a sample of 334,957 American university and college students, we compared MH comorbidities of 1885 GD individuals with an ED to demographically matched samples of non-GD individuals with an ED and GD individuals with no ED diagnosis. **Results:** Even following stringent demographic matching, GD participants with an ED had significantly more comorbid MH diagnoses (M = 4.23, SD = 2.23) than non-GD individuals with an ED (M = 2.86, SD = 1.98). Similarly, GD participants with an ED had significantly higher MH comorbidity than demographically matched GD individuals without an ED (M = 1.96, SD = 1.84). In GD individuals, ED diagnosis was associated with increased odds of experiencing minority stress events (OR: 2.19 95% CI [1.91–2.51]) and associated distress (OR: 2.17 95% CI [1.89–2.50]). **Conclusions:** We find that GD individuals with an ED report significantly elevated MH comorbidity, including serious MH disorders. Prompt intervention and proactive screening have an important role to play in supporting young adults in this vulnerable population.

## 1. Introduction

Eating disorders (EDs), including Anorexia Nervosa (AN), Bulimia Nervosa (BN) and Binge Eating Disorder (BED) [1], are psychological disorders in which an individual manipulates their dietary intake as a way to cope with difficult situations and emotions [2]. The prevalence of EDs is increasing, with a lifetime prevalence estimated at 3.3–18.6% for women and between 0.8 and 6.5% for men [3]. EDs are associated with significant mental health (MH) comorbidity, with over 70% of people with an ED reporting additional psychiatric diagnoses [4], including mood (43%) and anxiety (53%) disorders [5]. Recent systematic reviews have also found evidence of significantly elevated rates of Attention Deficit Hyperactivity Disorder (ADHD) [6], Bipolar Disorder (BD) [7] and Personality Disorder (PD) [8] in clinical ED populations.

Long-term health outcomes for individuals with an ED are poor: between 30% [9] and 64% [10] of individuals with an ED continue to meet diagnostic criteria 10 years after the initial diagnosis, and individuals with an ED report significantly lower quality of life [11] and impaired social functioning [12]. Moreover, ED-associated mortality is high: individuals with an ED are five times more likely to attempt suicide [13], whilst AN has the highest mortality rate of any MH disorder, with a weighted annual mortality rate of 5 per 1000 person-years [14]. ED treatment outcomes are poorer for individuals with MH comorbidities [15]. MH comorbidity is associated with poorer quality of life [16] and excess mortality [17] in individuals with an ED. Consequently, there is considerable interest in understanding the association between ED diagnosis and additional MH diagnoses that may impact treatment outcomes and quantity of life.

Gender-diverse (GD) individuals have a gender identity that does not match their birth-assigned sex [18]. GD individuals may have a binary transgender identity: trans women identify as female-gendered, while trans men identify as male [19]. Other GD individuals may have a non-binary identity, meaning they do not identify specifically with one gender [20]. GD people often experience gender dysphoria [18], a marked discrepancy between their experienced gender and their biological sex, resulting in significant psychological distress and impaired social and occupational functioning [1].

Compared with cisgender individuals, whose gender identity aligns with their birth sex, being GD is associated with an increased risk of disordered eating [21,22] and ED diagnosis [23]. In GD youth, prevalence estimates for clinical ED diagnosis range from 2% to almost 18% [23], compared to an estimated 5.7% in a general adolescent population [3]. ED symptomatology is also increased in GD youth, particularly when compared to cisgender males [24,25,26,27].

The reasons for this elevated ED symptomatology in GD populations are not fully understood. However, it is hypothesised that it may be explained by body image concerns: GD individuals have significantly more negative body image than their cisgender counterparts [28,29] due to the perceived incongruence between their physical appearance and gender identity [30,31]. Body image disturbance is associated with ED development [32,33] and so may mediate the relationship between GD identity and elevated ED psychopathology [23,34].

In addition to increased ED symptomatology, GD adolescents are at higher risk of other MH conditions [35], with GD children and adolescents presenting with elevated levels of MH comorbidity [36]. Over half of GD youth presenting to a US gender clinic reported clinically elevated symptoms of depression and anxiety [37], while a recent meta-analysis reported that 28.2% of GD youth had a lifetime history of non-suicidal self-injury, and 14.8% had attempted suicide [38]. Rates of Autism Spectrum Disorder (ASD) [39] and substance misuse [40] are also elevated in GD adolescents.

Furthermore, GD individuals with an ED diagnosis may be at even higher risk of MH comorbidities than GD individuals without an ED, including anxiety, depression and PTSD [41,42]. Moreover, GD individuals with an ED report strikingly high suicidality: Among GD youth with an ED, 74.8% reported a recent suicide attempt, compared to 25.9% of non-transgender females and 40.4% of non-transgender males with an ED [43].

These findings suggest that being GD and having an ED may have a compounding effect on MH [41], leaving GD individuals with an ED at extremely elevated risk of additional serious MH concerns [43]. However, to date, studies investigating MH comorbidity of ED diagnosis in GD individuals have been restricted to examination of individual diagnoses. This limits interpretation of findings, as MH diagnoses are highly comorbid [44], so examining bivariate associations alone may obscure true causative relationships [45]. Moreover, psychiatric multimorbidity iteself has been proposed as driver of poor health outcomes [46,47] and suicidality [48]. To our knowledge, no large-scale prior studies have systematically evaluated elevated risk of MH comorbidity in GD individuals with an ED. Moreover, previous studies have not concurrently controlled for demographic differences that may contribute to elevated MH comorbidity in GD individuals with an ED. Therefore, it is unclear whether associations between ED diagnosis, GD identity and comorbid MH diagnoses are true relationships or whether they are secondary to differing baseline symptomatology associated with underlying demographic differences.

In addition to characterising MH risks for GD individuals, it is important to explore the underlying contributors to elevated MH symptomatology in GD populations. Minority stress [49,50] is a well-established explanatory framework that conceptualises the relationship between social stressors and mental health difficulties in minoritised populations [51]. It has been extended through the Gender Minority Stress Framework to characterise unique stressors associated with being GD [52]. Within the Gender Minority Stress framework, gender minority individuals experience an increased burden of social stressors, including gender-based victimisation, rejection and discrimination, leading to an increased risk of developing MH difficulties [52,53].

There is considerable evidence that Gender Minority Stress is associated with increased MH difficulties in GD individuals [54,55]. Moreover, evidence suggests that Gender Minority Stress is associated with increased ED symptomatology [54], including binge eating, disordered weight control and poor body image [55] in GD individuals. However, despite the association between Gender Minority Stress and both mood disorders and ED symptomatology, to date, no study has investigated the relationship between Gender Minority Stress and MH comorbidity in GD individuals with an ED diagnosis.

This study aims to understand the relationship between gender diversity, ED diagnosis and MH comorbidity. We compare the total number of diagnosed MH conditions between GD individuals with an ED and comparator groups. This allows us to look at multimorbidity, which is associated with poor outcomes and suicidality, independently of specific diagnoses. We use propensity score matching (PSM) to robustly control for demographic differences between GD individuals with an ED and comparator groups in a large sample of American college students. This allowed us to stringently compare rates of MH comorbidity in GD individuals with an ED and demographically matched samples of GD individuals with no ED and non-GD individuals with an ED. Our primary hypotheses were that, even following stringent demographic matching,

- GD individuals who have an ED diagnosis would have an increased rate of comorbid MH diagnoses compared to non-GD individuals with an ED diagnosis;
- GD individuals who have an ED diagnosis would have an increased rate of comorbid MH diagnoses compared to GD individuals with no ED diagnosis.

We then completed exploratory analyses to explore associations between GD identity, ED diagnosis and a broad range of specific MH comorbidities in our demographically matched groups. This was to understand whether particular MH diagnoses were associated with observed differences in MH comorbidity between the groups and to begin to characterise the complex landscape of MH multimorbidity in these vulnerable populations.

We further hypothesized that, in individuals identifying as GD, even following demographic matching,

- ED diagnosis would be associated with greater likelihood of minority stress experiences;
- ED diagnosis would be associated with greater likelihood of perceived distress associated with minority stress experiences.

In an exploratory step, we also investigated the relationship between individual Gender Minority Stress events, including experiences of bullying, cyberbullying, sexual harassment, discrimination and microaggression, and ED diagnosis for GD individuals.

## 2. Materials and Methods

### 2.1. Data Source and Sample

Participant data were derived from the Fall 2019–Fall 2022 cohorts of the American College Health Association’s National College Health Assessment survey (NCHA III). This is an online self-report survey completed annually by approximately 100,000 students in twice-yearly cohorts [56]. Survey participants are drawn from more than 740 participating public and private educational institutions, including both 2- and 4-year colleges. The survey includes extensive questions about students’ health behaviours, including physical and mental health conditions, plus detailed demographic data including gender identity, birth sex, age, ethnicity and socio-economic indicators. During the 4.5-year span encompassed in our study, the ACHA-NCHA survey received 334,957 responses. These participants formed the full dataset from which our analytical sample was constructed.

### 2.2. Measures

#### 2.2.1. Dependent Variable: Number of Mental Health Conditions

Data about comorbid MH diagnoses was derived from the question “Have you ever been diagnosed by a healthcare or MH professional with any of the following ongoing or chronic conditions?” Answer categories were “Yes”, “No” and Not answered for each condition. “Yes” Answers to these questions for MH fields were combined into a numerical variable counting the total number of MH diagnoses, excluding ED diagnoses. The count of total MH diagnoses was used as we wanted a variable that captured multimorbidity and was agnostic to specific diagnostic categories. Where answers for more than two MH conditions were missing, the total number of MH conditions was also treated as missing. If only one or two items were not answered, the number of additional MH diagnoses was counted from the number of “Yes” answers. The question included an “Other” box with an option to write-in any additional diagnoses. Write-in answers were screened and categorised according to the prescribed categories or added to an “Other MH condition” category. Write-in answers that indicated suspected or self-diagnosed conditions were not included. However, although the wording of this question included reference to clinical diagnoses, it is not possible to determine whether participants included self-diagnosed conditions, except where write-in information was provided.

#### 2.2.2. Independent Variables

ED status was derived from the question “Have you ever been diagnosed by a healthcare or MH professional with any of the following ongoing or chronic conditions?” Participants choosing “Yes” for “Eating Disorders” were considered to have an ED. Details of specific ED diagnoses was not available from the survey. Moreover, although the question refers to clinical diagnosis, it was not possible to determine whether participants included self-diagnoses. Participants who did not answer this question were excluded.

GD status was derived from the questions“What sex were you assigned at birth?”, “Do you identify as transgender?” and “Which term do you use to describe your gender identity?” Diverse gender identities include trans man/woman, non-binary, agender, genderqueer and genderfluid. Participants were classed as GD if they endorsed a diverse gender identity from the suggested list of terms or from a write-in option; if they identified as transgender; or if they chose a gender identity that did not align with their birth sex. Write in options were screened and participants who gave ironic or offensive identities or stated that they did not endorse the concept of gender identity were not counted as GD. Participants were not included in the analytic sample if they did not answer any of these questions in a manner that allowed them to be identified as gender-diverse or not. For further details, see Supplementary Methods.

#### 2.2.3. Additional Demographic Variables

Participants’ birth sex was derived from the question “What sex were you assigned at birth?” Answer options included male, female, and intersex. Participants who chose intersex were excluded from further analysis as numbers in some cells were too small (n<10) for either regression analysis or matching (see Table 1). Participant age in years was extracted from answers to the question “How old are you?”

Participants’ socio-economic status was estimated from a question about parental education. This was extracted from participants’ answer the question “What is the highest level of education completed by either of your parents (or guardians)?” Answer categories included “Did not finish high school”, “High school diploma or GED”, “Attended college but did not complete degree”, “Associate’s degree”, “Bachelor’s degree”, “Master’s degree”, “Doctoral or professional degree” and “Don’t know”. Participants answering “Don’t know” were re-coded as belonging to the modal category (“Bachelor’s degree”). These categories were pooled into three larger categories: post-graduate education (master’s degree and doctoral degree); graduate education (bachelor’s degree and associate’s degree); or no college education (incomplete college degree; high-school or GED; and below high school) to create a Parent Education Level (PEL) variable.

Participants’ ethnicity was extracted from the question “How do you usually describe yourself?” which had the following fields: American Indian or Native Alaskan; Asian or Asian American; Black or African American; Hispanic or Latino/a/x; Middle Eastern/North African (MENA) or Arab Origin; Native Hawaiian or Other Pacific Islander Native; White; Biracial or Multiracial and Other. Due to small numbers, the “American Indian or Native Alaskan,” “MENA,” and “Native Hawaiian or Other Pacific Islander Native” options were collapsed into the Other category. Participants choosing multiple answers were also combined the “Biracial or Multiracial” category. If no category was selected, ethnicity was coded as missing.

#### 2.2.4. Minority Stress Experiences

Minority stress experiences were derived from the question “Within the last 12 months, have you had problems or challenges with any the following?” Relevant answer fields comprised bullying, cyberbullying, microaggression, sexual harassment and discrimination. Participants who endorsed experiencing any of these events were classified as having a minority stress experience. If one answer was omitted, this variable was constructed based on the questions that were answered. If more than one answer was missing, the variable was coded as missing. We constructed similar binary variables to track experiencing any of the five individual minority stress events.

Subjective distress associated with minority stress experiences was derived from the question “Within the last 12 months, to what extent did the following issue(s) cause you distress?” which is asked as a follow-up for each stressor identified and answered using a 4-point Likert scale. Following the dichotomising process used for similar stress [57] variables from the NCHA III dataset, we recoded participant responses as a binary variable representing the highest level of distress experienced across any event experienced. Answer categories “No distress” and “Minimal distress” were coded as not experiencing distress; “Moderate distress” and “High distress” were coded as experiencing distress. We also construct binary variables associated with each specific event type to investigate impact of subjective distress associated with different stressors.

### 2.3. Data Processing and Analytic Strategy

#### 2.3.1. Data Cleaning and Preprocessing

Data cleaning and preprocessing was completed using custom scripts written in the Python programming language (version 3.4) [58]. Statistical analysis, PSM and multiple imputation (MI) were carried out using the R programming language (Version 4.2.2) [59]. Pre-processing and analysis scripts can be viewed on Github here: https://github.com/rebecca-murphy-ed/NCHA-analysis, accessed on 20 January 2026. Prior to conducting statistical analysis, we pre-registered our analytic plan with the Open Science Foundation [60]. Our initial analytic plan can be found here: https://osf.io/y8zk7, accessed on 20 January 2026. Where analyses differed from the pre-registered plan, this is identified below.

We constructed an analytic sample consisting of three groups (N = 30,277):

- Individuals who identify as GD, diagnosed with an ED (GD + ED group, *n* = 1948).
- Individuals who do not identify as GD, diagnosed with an ED (ED group, *n* = 14,869).
- Individuals who identify as GD, not diagnosed with an ED (GD group, *n* = 13,460).

For initial demographic evaluation, we classified individuals who do not identify as GD and who are not diagnosed with an ED as belonging to an Other group (*n* = 304,679).

In addition to the full analytic sample, we constructed a slightly smaller complete dataset, comprising individuals with no missing data (N = 29,515, 97.48% of the analytic sample):

- Complete GD + ED group (*n* = 1885).
- Complete ED group (*n* = 14,616).
- Complete GD group (*n* = 13,014).

#### 2.3.2. Initial Demographic Evaluation

We computed summary statistics for all variables across all groups in the analytic sample. These were also compared to distributions in the Other group.

Initial imbalance between the GD + ED group and ED group was calculated for number of MH conditions and for baseline demographic variables: age, sex, ethnicity and parental SES. The GD + ED group and GD group were similarly compared. These groups were also compared to the Other group. Significant group differences in categorical variables were identified using a $\chi^{2}$ test; significant differences in continuous and count variables were identified using a *t*-test. Distributions of missing data were also computed at both a variable level and a case level for the analytic sample.

#### 2.3.3. Propensity Score Matching

We used propensity score matching (PSM) to construct a sampled subset of individuals with an ED that were demographically matched to the GD + ED group participants and a similarly matched group of GD individuals with no ED diagnosis.

Propensity score matching (PSM) was performed in the R programming language [61] using the MatchIt software package (version 4.5.4) [62]. The Complete ED group was matched to the Complete GD + ED group for the following variables: age, sex, ethnicity and parental SES. The matching algorithm used Nearest Neighbour propensity score matching without replacement. The propensity score was estimated using logistic regression to infer group status based on demographic covariates. This yielded a matched Complete ED group containing 1885 participants, with 12,731 participants discarded. Matching the Complete GD group to the Complete GD + ED group yielded a matched CompleteGD group containing 1885 participants with 11,129 participants discarded. Matching the Complete GD group also used Nearest Neighbour matching and yielded adequate balance between the groups.

#### 2.3.4. Statistical Analysis

Following calculation of descriptive statistics, a series of regression analyses were performed to test for statistically significant differences between the analytic groups. Initial analysis used the complete dataset, excluding participants with any missing variables. The same analysis was performed on the complete dataset following PSM.

First, a quasi-Poisson model was used to explore the relationship between analytic group (GD + ED vs. ED groups and GD + ED vs. GD group) and the number of comorbid MH conditions. A quasi-Poisson model was used because this distribution can model count data where overdispersion precludes accurate fitting using a Poisson model. Demographic covariates (age, birth sex, ethnicity and parental SES) were included as covariates both pre- and post-matching to account for residual variations. Categorical data with more than two values were dummy-coded using the modal category as the base category.

Exploratory analyses were then performed to understand the differences in specific mental health comorbidities between the analytic groups. A series of binary logistic regressions were performed to identify significant differences in diagnosis of specific MH conditions between individuals in the GD + ED group and the ED and GD groups. Similar logistic regression models were used to identify any differences in minority stress experiences and associated distress between the GD + ED group and the ED and GD groups.

#### 2.3.5. Sensitivity Analysis—Multiple Imputation of Missing Data

Data were missing from 2.52% of participants. As an additional sensitivity analysis, we used multiple imputation with chained equations (MI) to impute plausible missing values for participants with missing data from the full analytic sample. For full details, see the Supplementary Methods.

## 3. Results

### 3.1. Descriptive Analyses

#### 3.1.1. Participant Demographics

Table 1 shows the demographic characteristics of the total sample and the different participant groups. The majority of participants were white (56.1%) and had a female birth sex (67.4%) and female gender identity (64.3%). Of the 334,957 total participants, 16,817 (5.02%) reported a lifetime ED diagnosis and 15,408 (4.60%) identified as GD, with 1948 (0.58%) individuals endorsing both an ED diagnosis and being GD. Interestingly, although the largest single gender identity category endorsed by GD participants was non-binary, the majority of GD participants, both with (*n* = 1714, 88.0%) and without (*n* = 10,123, 75.2%) an ED, reported a female birth sex. The majority of participants reported that their parents had completed either an undergraduate or post-graduate degree qualification.

#### 3.1.2. Mental Health Diagnoses

Table 2 shows the number of MH diagnoses endorsed by survey participants, as well as the frequencies of individual diagnoses. The mean number of diagnoses identified by participants was 0.88, with 61.28% of participants reporting 0 MH diagnoses and the modal non-zero number of diagnoses being 1 (13.55% of participants). The most common diagnoses were anxiety and depression, endorsed by 29.54% and 23.47% of participants, respectively. The number of MH diagnoses was significantly increased (t = 137.18, *p* < 0.001) for individuals with an ED (M = 2.95, SD = 2.02) compared to those without (M = 0.77, SD = 1.28). MH diagnoses were also significantly increased (t = 76.32, *p* < 0.001) for individuals identifying as GD (M = 2.11, SD = 2.05) compared to those who did not (M = 0.82, SD = 1.34). Participants with an ED who identified as GD (M = 4.23, SD = 2.23) reported a significantly higher number of MH diagnoses (t = 26.93, *p* < 0.001) than individuals with an ED who were not GD (M = 2.78, SD = 1.93) and significantly more (t = 44.99, *p* < 0.001) MH diagnoses than GD individuals without an ED (M = 1.81, SD = 1.83).

### 3.2. Mental Health Comorbidities—Comparing GD and Non-GD Individuals with an ED Diagnosis

#### 3.2.1. Demographic Differences Between GD and Non-GD Individuals with an ED Diagnosis

**Figure 1:**
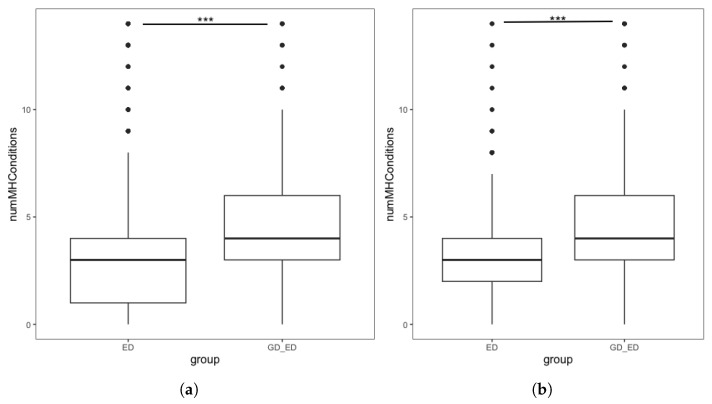
Boxplots showing the number of additional mental health conditions endorsed by individuals with an ED, split by GD status in the (**a**) complete dataset. Interquartile range: ED group [1, 4], GD + ED group: [3, 6] (**b**) Demographically matched sample from the complete dataset. Interquartile range: matched ED group [2, 4], GD + ED group: [3, 6]. *** denotes *p* < 0.001.

Our first hypothesis was that GD individuals with an ED diagnosis would have a greater number of comorbid MH diagnoses than individuals with an ED who were not GD. Initial analysis (Figure 1a) found significantly more additional MH diagnoses in the GD + ED group than the ED group. However, we also identified demographic differences between the two groups, with significant differences in birth sex, age and ethnicity (Table 3).

To reduce the confounding impact of these demographic differences, we repeated the analysis using the demographically matched ED group following PSM. Figure 1b shows the distribution in the number of MH diagnoses endorsed by individuals in the GD + ED and matched ED group. Initial analysis found that the number of additional MH conditions in the GD + ED group (M = 4.23, SD = 2.23) was significantly higher (t = 19.85, *p* < 0.001) than those in the matched ED group (M = 2.86, SD = 1.98). Quasi-Poisson regression found that for individuals with an ED, being GD was associated with a significantly higher number of additional MH diagnoses, confirming our hypothesis (Table 4).

This finding was maintained in two sensitivity analyses—one using the full analytic sample following multiple imputation of missing values and one using the complete dataset without PSM. This demonstrates that this finding was not due to biases introduced by using the complete dataset or by discarding unmatched participants following PSM (see Supplementary Results).

#### 3.2.2. Exploratory Analysis of Mental Health Diagnoses Associated with GD Status

GD status was associated with an increased likelihood of receiving any of the individual MH diagnoses considered, with most odds increasing approximately two-fold (see Table 5). Likelihood of receiving an ASD diagnosis was particularly elevated (OR 6.11, 95% CI [4.60–8.06]), with higher odds also seen for psychosis (OR 3.20, 95% CI [2.08–4.92]) and other MH difficulties (OR 6.11, 95% CI [2.33–4.21]).

### 3.3. Mental Health Comorbidities—Comparing GD Individuals with and Without an ED Diagnosis

#### 3.3.1. Demographic Differences Between GD Individuals with and Without an ED Diagnosis

**Figure 2:**
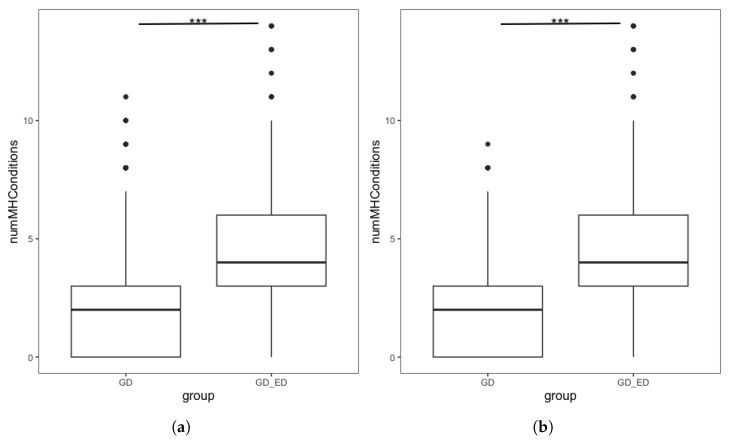
Boxplots showing the number of mental health conditions endorsed by GD individuals, split by ED status in (**a**) Complete dataset. Interquartile range: GD group [0, 3], GD + ED group: [3, 6] (**b**) Demographically Matched Sample from the Complete Dataset. Interquartile range: Matched GD group [0, 3], GD + ED group: [3, 6]. *** denotes *p* < 0.001.

Our second hypothesis was that GD individuals with an ED diagnosis would have a greater number of comorbid MH diagnoses than GD individuals without an ED. Initial analysis (Figure 2a) found significantly more MH diagnoses in the complete GD + ED group than the complete GD group. However, we also identified some demographic differences, with significant differences in birth sex and ethnicity (Table 6).

We repeated the analysis using the demographically matched GD group following PSM. Figure 2b shows the distribution in the number of MH diagnoses endorsed by individuals in the GD + ED and matched GD group. The number of additional MH conditions in the GD + ED group (M = 4.23, SD = 2.23) was significantly higher (t = −34.05, *p* < 0.001 than those in the matched GD group (M = 1.96, SD = 1.84). Quasi-Poisson regression found that for GD individuals, having an ED diagnosis was associated with a significantly higher number of additional MH diagnoses, confirming our hypothesis (Table 7). This finding was maintained in sensitivity analyses using the full analytic sample following MI and using the complete dataset without PSM (see Supplementary Results).

#### 3.3.2. Exploratory Analysis of Mental Health Comorbidities Associated with ED Diagnosis

ED diagnosis was associated with an increased likelihood of receiving any of the individual MH diagnoses considered, with a minimum two-fold increase in odds of receiving a specific diagnosis (see Table 8). Odds of several MH diagnoses, including addictions, anxiety, depression gambling disorder, PDs and psychosis, were over five times higher for GD individuals with than without an ED diagnosis.

### 3.4. Minority Stress Experiences in GD Individuals with and Without an ED Diagnosis

Our third and fourth hypotheses were that minority stress experiences and associated distress would be associated with ED diagnosis in GD individuals. For GD individuals, ED diagnosis was associated with a two-fold increase in odds of experiencing any minority stress event, and of experiencing associated distress (see Table 9). As a further exploratory analysis, we investigated the relationship between specific stressor events and ED diagnosis in GD individuals. Odds of experiencing any of the minority stress events considered were significantly increased when individuals had an ED diagnosis.

## 4. Discussion

### 4.1. Relationship Between Gender Diversity and MH Comorbidities in Individuals with an ED

This study demonstrates a very high prevalence of multiple comorbid MH diagnoses in GD individuals with an ED. Consistent with our first hypothesis, we found that GD individuals with an ED diagnosis have significantly more comorbid MH diagnoses than non-GD individuals with an ED diagnosis. This is consistent with prior findings of increased likelihood of mood disorders [41] and significantly increased incidence of suicidality and deliberate self-injury [43] in GD individuals with an ED.

It is possible that the elevated comorbidity of ED diagnosis in GD individuals simply represents the additive effect of higher MH comorbidity in both GD individuals [35,36] and individuals with an ED diagnosis [4]. However, it is also possible that the increased comorbidity is associated with a further common factor, such as underlying psychological distress [45], propensity towards MH difficulties [63] or historic trauma experience [64], which is associated with many MH diagnoses. Moreover, although PSM allowed us to control for measured demographic differences between GD and non-GD individuals with an ED, there may be other unmeasured differences, such as historical trauma experience, that account for the underlying differences between the groups. One of the limitations of using the total number of diagnoses as our outcome variable, as this removes information about the co-association of multiple diagnoses. Consequently, we were not able to explore patterns of comorbidity and how these differ between our analytic groups.

Also of note is the very high prevalence of birth females in the GD + ED group. Only a minority (8.06%) of participants in this group endorsed a female gender identity, with the largest single identity category being non-binary (45.1%). However, when birth sex was considered, the overwhelming majority (88.0%) of individuals in the GD + ED group were female. The majority of those in the ED group also had a female birth sex (92.1%), suggesting that this is reflective of an overall high prevalence of EDs in birth females. This may provide a rationale for earlier findings that prevalence of ED symptomatology among GD youth was similar to cisgender female peers [65,66], as the majority of GD participants in our study shared with cisgender females the characteristic of being birth females and may draw on similar ED behaviours to manage distress regarding their physical appearance. This highlights the importance of recording both gender identity and birth sex for clinical and research purposes, as lacking this information makes it challenging to characterise the experiences and clinical needs of GD individuals.

Overall, these findings highlight the importance of gender diversity awareness for clinicians working in ED services, as GD individuals within this clinical population are at increased risk of significant psychological distress and associated psychiatric comorbidity. The high level of comorbidity in this group also highlights the need for integrated, person-centred models of care that holistically address all of an individual’s difficulties [46].

Our exploratory analysis of specific MH disorders in GD individuals with an ED identified an increased risk of all diagnoses considered. Incidence of several common MH disorders, including anxiety (89.39%) and depression (87.59%), was strikingly high in the GD + ED group when compared with the ED group, with the majority of participants endorsing both comorbid diagnoses. This finding is notable as it highlights the further increased risk of mood disorders in GD individuals with an ED, even when compared to the high comorbidity in clinical ED populations [4].

Exploratory analysis further identified significantly higher odds of ASD (OR: 6.11, 95% CI [4.6, 8.06]) in GD compared to non-GD individuals with an ED. This is consistent with prior findings of significantly elevated odds of neurodevelopmental diagnoses in GD populations [67]. A recent meta-analysis has found significant co-occurrence of Avoidant/Restrictive Food Intake Disorder (ARFID) in individuals with ASD [68]. Moreover, prior research has identified significantly elevated ARFID symptoms in GD individuals, with 75% of GD participants in one survey screening positive for ARFID [69], whilst GD individuals with an ED were less likely to receive a diagnosis of a restrictive ED [41]. It is possible that the higher proportion of individuals with an ASD diagnosis in our GD + ED group is similarly associated with elevated rates of ARFID diagnoses. However, we were not able to explore this possibility, as the lack of specific diagnostic information in our dataset precluded this analysis.

### 4.2. Relationship Between ED Diagnosis and MH Comorbidities in GD Individuals

Our second hypothesis was that GD individuals with an ED diagnosis would have an increased risk of comorbid MH diagnoses compared to GD individuals with no ED diagnosis. Consistent with this hypothesis, we found that GD individuals with an ED diagnosis have significantly more comorbid MH diagnoses than GD individuals without an ED. Odds ratios for depression (OR: 6.67, 95% CI [5.65, 7.88]) and anxiety (OR: 7.16, 95% CI [5.96, 8.51]) identified significantly elevated co-occurrence of these diagnosis in the GD + ED group compared to GD individuals without an ED diagnosis, with the majority of individuals receiving both diagnoses. Moreover, odds ratios for a number of serious MH conditions, including psychosis (OR 6.80, 95% CI [3.82, 12.06]), addictions (OR: 5.82, 95% CI [3.92, 8.63]) and PTSD (OR: 4.83, 95% CI [4.14, 5.64]), were also strikingly high. These findings highlight that an ED diagnosis is an indicator of significant psychological distress in GD individuals. Although ED symptomatology and disordered eating behaviours are associated with body dissatisfaction in GD individuals [70], it is important that ED symptomatology is not treated as secondary to gender dysphoria: Given the high prevalence of ED symptomatology in GD individuals, this suggests a need for routine screening for ED difficulties in gender medicine settings and highlights the importance of prompt access to specialist treatment where clinical eating difficulties are identified [71].

It is possible that the very high rates of MH comorbidity, including serious MH conditions, may explain the highly elevated rates of suicidality and suicide attempts observed in GD individuals with an ED [43]. Research into suicidal behaviour [45] has found that although suicidal ideation is strongly associated with depression, progression to suicide attempts is associated with MH diagnoses associated with anxiety and agitation, for example, PTSD, and impulsivity and poor impulse control (including addictions and conduct disorder). The elevated rates of these disorders in our GD + ED group suggest that serious MH comorbidity, or the presence of multimorbidity, may mediate the observed relationship between ED diagnosis and increased suicidality in GD populations. We were not able to test this hypothesis as information on suicidality was not included in the data we requested from the NCHA.

GD individuals can lack access to appropriate healthcare and may seek treatment later than non-GD individuals [72]. Consequently, it is possible that the observed differences in comorbidity between the GD + ED and GD groups reflects differential access to healthcare and formal diagnosis, such that the lower comorbidity rate in the GD group underestimates true clinical need. This highlights the importance of access to appropriate clinical care for GD individuals when MH needs are identified.

### 4.3. Relationship Between ED Diagnosis and Minority Stress Events in GD Individuals

Prior research has found that Gender Minority Stress is associated with both MH difficulties generally [54,55] and ED symptomatology specifically [25,73] in GD individuals. Given these findings, we wondered whether minority stress experiences could be related to elevated rates of ED diagnosis in GD individuals. Our final two hypotheses were that in GD individuals, an ED diagnosis would be associated with both an increased likelihood of experiencing minority stress events and increased distress associated with minority stress experiences. Consistent with these hypotheses, we found significantly higher odds of experiencing any minority stress event in GD individuals with an ED diagnosis. We also found significantly higher odds of feeling distress associated with these events. When we examined individual stressor experiences, we found similarly increased odds of experiencing all types of stressor events.

**Figure 3:**
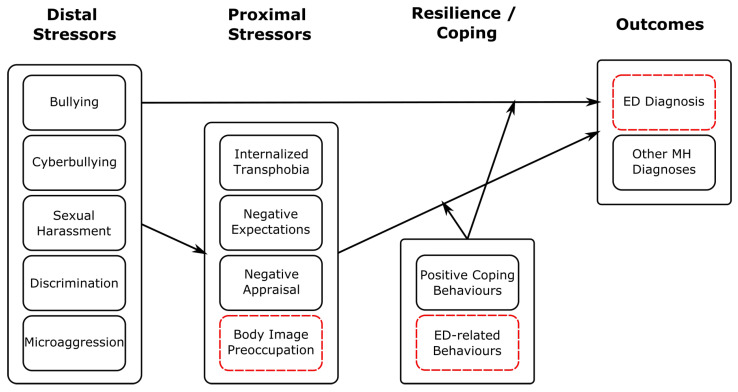
Schematic illustrating how our findings regarding stressor events fit within the Gender Minority Stress model. Here, distal stressors comprise those considered in our study. We have added ED-specific stressors, coping behaviours and outcomes highlighted with red, dashed boxes. This is a descriptive figure designed to show how our findings may be consistent with a minority stress framework; relations shown have not been tested empirically here.

The stressor events that we considered (bullying, cyberbullying, sexual harassment, discrimination and microaggression) can all be considered distal stressors within the Gender Minority Stress model [52]. Consistent with earlier findings [55], these stressors were all associated with ED diagnosis in GD individuals. As illustrated in Figure 3, this could support the minority stress hypothesis that elevated rates of distal stressor events potentiate both proximal stressors and elevated risk of MH difficulties includeing EDs. Moreover, as found in earlier studies [42], rates of PTSD diagnosis in GD individuals with an ED were very high (48.38%). This may be consistent with a relationship in which stressor events are associated with psychological distress and managed through a range of coping strategies, including disordered eating behaviour, increasing risk of ED diagnosis.

However, it is important to note that given the cross-sectional design of our study we cannot draw any conclusions regarding the temporal relationship between minority stress experiences and ED symptomatology. Although our findings are consistent with a relationship in which minority stress experiences lead to increased distress and increased likelihood of ED diagnosis, it is equally plausible that pre-existing psychological vulnerability, associated with an ED diagnosis, leads to increased risk of stressor experiences [74] or to negative appraisal of neutral social events [75], which are then interpreted as discriminatory.

We also found that GD individuals with an ED reported increased distress associated with minority stress experiences. There are several possible interpretations of this finding. It is possible that this increased distress indicates increased severity of the minority stress experience, such that more significant incidents, triggering greater distress, are associated with increased likelihood of clinical ED symptomatology. An alternative interpretation is that increased distress is associated with a more negative subjective appraisal of an experience [76]. Within this interpretation, increased distress could indicate an increase in proximal stressors, including negative expectations of future events and internalised self-blame, both of which have been associated with particularly high MH comorbidity in GD individuals [55]. As negative social appraisal is also found in individuals with an ED [75], it may be that this altered social cognition associated ED diagnosis is responsible for increased subjective experience of distress. Unfortunately, the cross-sectional nature of our study does not allow us to distinguish between these possibilities.

### 4.4. Strengths and Limitations

This study has several strengths. Firstly, due to the high number of participants in the NHCA-ACHA survey, we were able to include data from over 1000 participants who both identified as GD and had received an ED diagnosis, despite the relative rarity of both conditions. This ensured that the size of our analytic sample had sufficient statistical power to give confidence in our ability to detect an effect of this size. Moreover, the large number of participants also enabled us to use PSM to generate a demographically matched comparison groups. As PSM requires discarding unmatched participants from analysis, we completed a further sensitivity analysis to ensure that this did not introduce bias into our results. This further increases confidence in our findings, as PSM reduces the likelihood that differences in MH diagnoses are caused by demographic differences between participant groups and our findings were maintained across the sensitivity analysis. A further strength of this study is our use of a clear, pre-registered analytic plan for statistical analysis of our data. Our use of MI to account for missing data demonstrates that our findings are robust and not influenced by biases in the underlying dataset.

However, it is also important to acknowledge the limitations of our findings. Firstly, although the NCHA survey asks broadly about ED diagnosis, it does not record information about specific diagnoses, for example, AN or BN. Therefore, it was not possible for us to differentiate between different EDs experienced by the participants. This is a significant limitation, as although there are cross-diagnostic commonalities between different EDs [77], they may have different comorbidity profiles [78]. Moreover, this is of particular salience in GD populations, where different disordered behaviours, such as restriction, which are associated with particular ED diagnoses, may be specifically motivated by attempts to alleviate gender dysphoria [24]. As we did not have access to fine-grained diagnostic information, we were not able to explore this further. Similarly, given earlier findings that ARFID symptoms are significantly elevated in GD youth [69] and that ARFID symptomatology is associated with ASD [68], it is possible that a more fine-grained analysis of ED diagnoses would find an association between ASD and ARFID in GD individuals. However, we were also unable to explore this.

A limitation in our statistical analysis is the use of the OR to report results of logistic regression analyses into MH comorbidities. When the base rate of diagnoses is very low, the OR closely approximates the relative risk ratio. However, in our dataset, the rate of reported diagnoses of many MH conditions was extremely high across all analytic groups. Under these conditions, the OR is inflated relative to the relative risk [79,80] as the rate of chance co-occurrence is increased. This makes it challenging to distinguish between true comorbidity clustering and coincidental co-association.

Thirdly, our analysis relies on self-reported clinical MH diagnoses, which may be affected by recall bias. Furthermore, although the survey asks specifically about clinical diagnosis, it was clear from write-in answers that a few participants were including self-diagnosed or suspected conditions, in addition to formal clinical diagnoses. Although explicitly acknowledged self-diagnoses were not included in the analysis, it was not possible to identify these where no additional write-in information was provided. Therefore, it is possible that the number of different MH conditions endorsed by participants may not be accurately reported. For example, participants may have included historic diagnoses or reported misremembered or poorly understood clinical discussions. This is of particular relevance as the number of MH conditions endorsed by participants is strikingly high. Conversely, use of formal diagnoses may underestimate psychological difficulties, particularly in individuals from marginalised or minoiritsed communities who may have lacked access to appropriate healthcare services [72,81]. GD individuals face increased barriers to accessing medical services [72]. They may also be at risk of underdiagnosis of additional mental health needs due to over-focus on their gender dysphoria [71]. This suggests that our analysis may differentially underestimate the MH comorbidities of GD relative to non-GD participants.

A further limitation is that schools and colleges self-select to participate in the NCHA-ACHA Survey. Moreover, although individuals at participating institutions are invited at random to complete the survey, neither invitations nor responses are weighted to ensure that respondents are representative of the college population or national demographics. This is evidenced by the high percentage of participants with parents educated to degree graduate or post-graduate level. This limits the generalisability of our findings, as respondents are a self-selecting group of college students, whose difficulties may not be typical of the wider American population, including non-student populations or older Americans. As the cohort is derived entirely from American colleges, findings may not generalise to non-American contexts. Moreover, survey data were from the Fall 2019–Fall 2022 cohorts of the NCHA III, which includes the period of the Covid pandemic. This further limits generalisability of our findings, as this period is associated with changes in ED symptom severity and treatment access [82].

Finally, as our analysis uses survey data, we can only draw conclusions about the association between a GD identity, ED diagnosis, other MH diagnoses and minority stress experiences. Although our findings are consistent with ED diagnosis increasing vulnerability to additional MH comorbidities in GD individuals, it is also possible that increased MH vulnerability is a driver of increased ED risk. Similarly, although minority stress experiences may lead to increased ED risk, the converse relationship in which altered cognition and behaviour associated with an ED diagnosis lead to increased vulnerability to minority stress events. As we cannot infer any causal relationships, it is not possible to disambiguate these scenarios.

#### Clinical Implications and Future Directions

Our findings have implications for both clinical practice and future research. We find that MH comorbidity and incidence of additional diagnoses of severe mental illness are highly elevated GD young adults with an ED diagnosis. This suggests that an ED diagnosis may be associated with significant underlying psychological distress in GD individuals and that prompt access to specialist MH care is necessary to support wellbeing. This is of particular relevance, as concerns have been raised that diagnostic overshadowing can lead to under-treatment of MH difficulties in GD populations [71].

Due to the elevated prevalence of ED symptomatology in GD individuals, ED screening may be indicated in gender services to identify individuals with clinical eating difficulties and enable prompt referral to specialist treatment services. We further suggest that, where clinically significant eating difficulties are identified in GD individuals, screening for additional signs of psychological distress [83] may help to identify further need for specialist intervention and guide individual treatment goals.

Finally, given the highly elevated number of comorbid MH diagnoses received by GD individuals with an ED, further research is warranted to understand the development and interrelation of these difficulties in this vulnerable population. Exploration of the association between MH comorbidity and specific ED diagnoses is also of considerable interest, given the known associations between ARFID, ASD and gender identity [69]. Given the extremely high prevalence of suicidal ideation and suicide attempts found in this group [43], it is also of considerable clinical importance both to understand the relationship between multimorbidity and suicidality; to understand whether particular MH difficulties have a mediating effect; and to develop effective, person-centred interventions that holistically address the underlying causes of this significant psychological distress.

## 5. Conclusions

Our study found that GD individuals with an ED had significantly more comorbid MH conditions than both non-GD individuals with an ED and GD individuals without an ED. The odds of these individuals having several serious MH comorbidities were markedly elevated. This suggests that this population may benefit from proactive screening and rapid access to specialised treatment pathways. We further found that among GD individuals, ED diagnosis was associated with increased odds of experiencing minority stress events and associated distress. These findings are consistent with the Gender Minority Stress model, in which minority stress experiences may potentiate risk of developing a range of MH conditions, including EDs, in GD populations.

## Figures and Tables

**Table 1:** Demographic characteristics of participants in the Fall 2019–Fall 2022 cohorts of the NCHA III Survey. Abbreviations: PEL—Parental Education Level. Note that individuals who do not identify as GD and have chosen an ”Other” gender identity either used the write-in box to state that they did not endorse the concept of gender identity or left the write-in box blank.

	GD + ED Group	ED Group	GD Group	Other Group	Total
* n *	1948	14,869	13,460	304,679	334,957
Age M (SD)	22.0 (5.37)	22.6 (6.02)	22.2 (5.67)	23.1 (6.66)	23.1 (6.59)
Birth Sex *n* (%)					
Female	1714 (87.99)	13,701 (92.14)	10,123 (75.21)	200,059 (65.66)	225,597 (67.38)
Male	204 (10.47)	1142 (7.68)	3222 (23.93)	101,424 (33.29)	105,993 (31.66)
Intersex	23 (1.18)	5 (0.03)	66 (0.49)	30 (0.01)	124 (0.037)
Missing	7 (0.36)	21 (0.14)	49 (0.36)	3166 (1.04)	3243 (0.97)
Gender Identity *n* (%)					
Female	157 (8.06)	13,663 (91.90)	1879 (13.98)	199,770 (65.57)	215,469 (64.34)
Male	108 (5.54)	1126 (7.57)	1291 (9.60)	100,961 (33.14)	103,486 (30.90)
Non-binary	878 (45.40)	0 (0.00)	5013 (37.30)	0 (0.00)	5891 (1.75)
Genderqueer	201 (10.34)	0 (0.00)	1337 (9.93)	0 (0.00)	1538 (0.46)
Genderfluid	202 (10.49)	0 (0.00)	1304 (9.69)	0 (0.0)	1506 (0.45)
Other	107 (5.49)	48 (0.32)	716 (5.32)	541 (0.18)	1412 (0.42)
Trans Man	152 (7.80)	0 (0.00)	800 (5.94)	0 (0.00)	952 (0.28)
Agender	96 (4.93)	0 (0.00)	694 (5.16)	0 (0.00)	790 (0.24)
Trans Woman	40 (2.05)	0 (0.00)	391 (2.90)	0 (0.00)	431 (0.13)
Intersex	6 (0.31)	0 (0.00)	24 (0.18)	0 (0.00)	30 (0.0090)
Missing	1 (0.051)	32 (0.22)	11 (0.082)	3408 (1.12)	3452 (1.03)
Ethnicity *n* (%)					
White	1286 (66.38)	10,254 (68.99)	8127 (60.55)	168,330 (55.25)	187,997 (56.14)
Asian	106 (5.45)	945 (6.36)	1419 (10.53)	43,814 (14.38)	46,284 (13.82)
Multiracial	338 (17.56)	2033 (12.81)	345 (15.06)	33,122 (10.87)	37,407 (11.16)
Hispanic	111 (5.66)	1097 (7.38)	986 (7.33)	31,396 (10.87)	33,590 (10.03)
Black	42 (2.16)	292 (1.96)	502 (3.73)	15,736 (5.16)	16,572 (4.95)
Other	53 (2.72)	304 (2.04)	311 (2.31)	7767 (2.55)	8435 (2.52)
Missing	5 (0.26)	70 (0.47)	82 (0.61)	4515 (1.48)	4672 (1.39)
PEL *n* (%)					
Graduate	720 (36.96)	5395 (36.28)	4959 (36.84)	117,022 (38.41)	128,096 (38.24)
Postgraduate	766 (39.32)	6010 (40.42)	5171 (38.42)	104,564 (34.32)	116,511 (34.78)
No college	459 (23.56)	3424 (23.03)	3265 (24.26)	78,954 (25.91)	86,102 (25.71)
Missing	3 (0.15)	40 (0.27)	65 (0.48)	4140 (1.36)	4248 (1.27)

**Table 2:** Mental health diagnoses endorsed by participants. The top three rows present information about the number of different MH conditions endorsed by participants. The remaining rows present the percentage of participants endorsing each specific disorder. Abbreviations: ADHD—Attention Deficit/Hyperactivity Disorder; ASD—Autism Spectrum Disorder; OCD—Obsessive Compulsive Disorder; PD—Personality Disorder; PTSD—Post-Traumatic Stress Disorder.

	GD + ED Group	ED Group	GD Group	Other Group	Total
Total diagnoses M (SD)	4.23 (2.23)	2.78 (1.93)	1.81 (1.83)	0.73 (1.22)	0.88 (1.40)
% No Diagnoses	2.76	11.69	35.78	65.23	61.28
Modal Diagnoses >0 (%)	4 (19.05)	2 (23.10)	2 (18.23)	1 (13.61)	1 (13.55)
ADHD *n* (%)	763 (40.48)	3464 (23.69)	2883 (22.11)	25,479 (8.63)	32,588 (10.04)
Addiction *n* (%)	165 (8.75)	762 (5.21)	232 (1.78)	3048 (1.03)	4207 (1.30)
Anxiety *n* (%)	1685 (89.39)	11,280 (77.18)	6731 (51.61)	76,227 (25.83)	95,923 (29.54)
ASD *n* (%)	333 (17.67)	454 (3.11)	973 (7.46)	3192 (1.08))	4952 (1.53)
Bipolar *n* (%)	354 (18.78)	1454 (9.95)	354 (5.18)	4658 (1.58)	7141 (2.20)
Depression *n* (%)	1651 (87.59)	10,251 (70.14)	6227 (47.75)	58,062 (19.67)	76,191 (23.47)
Gambling *n* (%)	43 (2.28)	112 (0.77)	18 (0.14)	177 (0.060)	350 (0.11)
Insomnia *n* (%)	705 (37.40)	3326 (22.76)	1527 (11.71)	13,653 (4.62)	12,911 (5.92)
OCD *n* (%)	696 (36.92)	3916 (26.79)	1223 (9.38)	10,110 (3.43)	15,945 (4.91)
PD *n* (%)	316 (16.76)	984 (6.73)	363 (2.78)	1828 (0.62)	3491 (1.08)
Psychosis *n* (%)	90 (4.77)	207 (1.42)	123 (0.94)	542 (0.18)	962 (0.30)
PTSD *n* (%)	912 (48.38)	3787 (25.91)	2005 (15.37)	14,143 (4.79)	20,847 (6.42)
Other MH *n* (%)	182 (9.66)	472 (3.22)	444 (3.40)	1870 (0.63)	2968 (0.91)

**Table 3:** Differences in MH comorbidities and demographic characteristics of GD and non-GD individuals with an ED diagnosis. Significant differences in MH diagnoses are found, as well as differences in age, birth sex and ethnicity. *** denotes *p* < 0.001.

	GD + ED Group		ED Group					
	M	SD	M	SD	* t *	Cohen’s d	* p *	
Total Diagnoses	4.23	2.23	2.79	1.93	−26.93	−0.70	<0.001	***
Age (years)	21.93	4.88	22.63	6.00	5.64	0.13	<0.001	***
	*n*	%	*n*	%	$\chi^{2}$	df	*p*	
Birth Sex					16.32	1	<0.001	***
Female	1691	89.71	13,506	92.41				
Male	194	10.29	1110	7.59				
Ethnicity					40.08	5	<0.001	***
White	1260	66.84	10,158	69.50				
Multiracial	329	17.45	1880	12.86				
Hispanic	106	5.62	1076	7.36				
Asian	102	5.41	926	6.34				
Black	40	2.12	285	1.95				
Other	48	2.55	291	1.99				
PEL					0.37	2	0.83	
Graduate	697	36.98	5324	36.43				
Post-graduate	751	39.84	5927	40.55				
No college	437	23.18	3365	23.02				

**Table 4:** Parameters of a quasi-Poisson regression model predicting number of MH comorbidities of GD and non-GD individuals with an ED diagnosis. GD status is a significant predictor of MH comorbidity. Model fitting was performed on a demographically matched sample from the complete dataset. Abbreviations: PEL—Parent Education Level. Note that categorical variables are dummy coded with the modal category as the reference category. Asterisks denote statistical significance: * *p* < 0.05, ** *p* < 0.01, *** *p* < 0.001.

Parameter	Coefficient	Standard Error	z Value	*p*	
Intercept	0.85	0.049	17.50	<0.001	***
Is GD	0.39	0.021	18.42	<0.001	***
Age (Years)	0.0087	0.0020	4.44	<0.001	***
Birth Sex (Male)	0.090	0.033	2.71	0.0068	**
PEL (Postgraduate)	−0.043	0.024	−1.78	0.074	
PEL (No College)	0.076	0.027	2.83	0.0047	**
Ethnicity (Multiracial)	0.053	0.027	1.96	0.049	*
Ethnicity (Hispanic)	−0.21	0.050	−4.23	<0.001	***
Ethnicity (Asian)	−0.085	0.048	−1.76	0.078	
Ethnicity (Black)	0.13	0.067	1.97	0.048	*
Ethnicity (Other)	0.0042	0.067	0.063	0.95	

**Table 5:** Descriptive statistics and parameter estimates from the logistic regression models exploring the relationship between gender diversity and MH diagnosis in individuals with an ED using the demographically matched sample of participants. All models included an intercept term and controlled for age, birth sex, ethnicity and parental education level to account for residual variance within the matched sample. Abbreviations: ADHD—Attention Deficit/Hyperactivity Disorder; ASD—Autism Spectrum Disorder; OCD—Obsessive Compulsive Disorder; PD—Personality Disorder; PTSD—Post-Traumatic Stress Disorder. Asteristks denote statistical significance: * *p* < 0.05, *** *p* < 0.001.

	GD + ED Group		ED Group					
MH Condition	*n*	%	*n*	%	OR	95% CI	*p*	
ADHD	763	40.48	475	25.20	2.02	[1.76, 2.32]	<0.001	***
Addiction	165	8.75	90	4.77	1.92	[1.46, 2.51]	<0.001	***
Anxiety	1685	89.39	1460	77.45	2.54	[2.11, 3.06]	<0.001	***
ASD	333	17.67	65	3.44	6.11	[4.6, 8.06]	<0.001	***
Bipolar	354	18.78	191	10.13	2.06	[1.70, 2.49]	<0.001	***
Depression	1651	87.59	1347	71.46	2.86	[2.41, 3.40]	<0.001	***
Gambling	43	2.28	23	1.22	1.93	[1.11, 3.36]	0.02	*
Insomnia	705	37.40	465	24.67	1.83	[1.59, 2.11]	<0.001	***
OCD	696	36.92	511	27.11	1.58	[1.38, 1.82]	<0.001	***
PD	316	16.76	138	7.32	2.57	[2.08, 3.18]	<0.001	***
Psychosis	90	4.77	30	0.95	3.20	[2.08, 4.92]	<0.001	***
PTSD	912	48.38	511	27.11	2.58	[2.25, 2.96]	<0.001	***
Other MH	182	9.66	62	3.29	3.13	[2.33, 4.21]	<0.001	***

**Table 6:** Differences in MH comorbidities and demographic characteristics of GD individuals with and without an ED Diagnosis. Significant differences in MH diagnosis were found, as well as demographic differences in birth sex and ethnicity. *** denotes *p* < 0.001.

	GD + ED Group		GD Group					
	M	SD	M	SD	*t*	Cohen’s d	*p*	
Total Diagnoses	4.23	2.23	1.81	1.83	−44.99	−1.19	<0.001	***
Age (years)	21.93	4.88	22.11	5.47	1.51	0.036	0.13	
	*n*	%	*n*	%	$\chi^{2}$	df	*p*	
Birth Sex					175.98	1	<0.001	***
Female	1691	89.71	9903	76.10				
Male	194	10.29	3111	23.91				
Ethnicity					76.09	5	<0.001	***
White	1260	66.84	7977	61.30				
Multiracial	329	17.45	1956	15.03				
Hispanic	106	5.62	963	7.40				
Asian	102	5.41	1347	10.35				
Black	40	2.12	486	3.73				
Other	48	2.55	285	2.55				
PEL					1.23	2	0.54	
Graduate	697	36.98	4829	37.11				
Post-graduate	751	39.84	5038	38.71				
No college	437	23.18	3147	24.18				

**Table 7:** Parameters of a quasi-Poisson regression model to predict the number of MH comorbidities of GD and non-GD individuals with an ED diagnosis. Model fitting was performed on a demographically matched sample from the complete dataset. ED status is a significant predictor of MH comorbidity. Abbreviations: PEL—Parent Education Level. Note that categorical variables are dummy-coded with the modal category as the reference category. Asterisks denote statistical significance: * *p* < 0.05, ** *p* < 0.01, *** *p* < 0.001.

Parameter	Coefficient	Standard Error	t	*p*	
Intercept	1.32	0.17	7.80	<0.001	***
Has ED	1.26	0.066	19.09	<0.001	***
Age-Years	0.032	0.0071	4.62	<0.001	***
Birth Sex-Male	−0.10	0.11	−0.95	0.34	
PEL-Postgraduate	−0.18	0.076	−2.34	0.019	*
PEL-No College	0.24	0.088	2.71	0.0067	**
Ethnicity-Multiracial	0.029	0.089	0.33	0.74	
Ethnicity-Hispanic	−0.70	0.0.15	−4.76	<0.001	***
Ethnicity-Asian	−0.34	0.15	−2.33	0.020	*
Ethnicity-Black	0.20	0.23	0.84	0.40	
Ethnicity-Other	0.0035	0.22	0.016	0.99	

**Table 8:** Descriptive statistics and parameter estimates from the logistic regression models exploring the relationship ED diagnosis and other MH diagnosis in GD individuals using the demographically matched sample of participants. All models included an intercept term and controlled for age, birth sex, ethnicity and parental education level to account for residual variance within the matched sample. Abbreviations: ADHD—Attention Deficit/Hyperactivity Disorder; ASD—Autism Spectrum Disorder; OCD—Obsessive Compulsive Disorder; PD—Personality Disorder; PTSD—Post-Traumatic Stress Disorder. *** denotes *p* < 0.001.

	GD + ED Group		GD Group					
MH Condition	* n *	%	* n *	%	OR	95% CI	* p *	
ADHD	763	40.48	450	23.87	2.17	[1.89, 2.50]	<0.001	***
Addiction	165	8.75	31	1.64	5.82	[3.92, 8.63]	<0.001	***
Anxiety	1685	89.39	1060	56.23	7.12	[5.96, 8.51]	<0.001	***
ASD	333	17.67	135	7.16	2.79	[2.26, 3.45]	<0.001	***
Bipolar	354	18.78	109	5.78	3.77	[3.01, 4.72]	<0.001	***
Depression	1651	87.59	986	52.32	6.67	[5.65, 7.88]	<0.001	***
Gambling	43	2.28	3	0.16	6.67	[5.65, 7.88]	<0.001	***
Insomnia	705	37.40	233	12.36	4.26	[3.61, 5.04]	<0.001	***
OCD	696	36.92	208	11.03	4.78	[4.03, 5.69]	<0.001	***
PD	316	16.76	67	3.55	5.51	[4.19, 7.25]	<0.001	***
Psychosis	90	4.77	14	0.74	6.80	[3.82, 12.06]	<0.001	***
PTSD	912	48.38	316	16.76	4.83	[4.14, 5.64]	<0.001	***
Other MH	182	9.66	64	3.40	3.04	[2.27, 4.07]	<0.001	***

**Table 9:** Frequency of experiencing minority stress events and parameter estimates from logistic regression models exploring the relationship ED diagnosis and minority stress experiences in GD individuals. All models included an intercept term and controlled for age, birth sex, ethnicity and parental education level to account for residual variance within the matched sample. *** denotes *p* < 0.001.

	GD + ED Group		GD Group					
	* n *	%	* n *	%	OR	95% CI	* p *	
Any Minority Stress	1369	72.63	1040	55.17	2.17	[1.89, 2.50]	<0.001	***
Any Distress	763	40.48	450	23.87	2.19	[1.91, 2.51]	<0.001	***
Bullying	348	18.46	199	10.56	1.91	[1.58, 2.31]	<0.001	***
Cyberbullying	240	12.73	127	6.73	2.01	[1.60, 2.52]	<0.001	***
Sexual Harassment	661	35.07	321	17.03	2.65	[2.27, 3.08]	<0.001	***
Discrimination	730	38.73	464	24.62	1.95	[1.69, 2.25]	<0.001	***
Microaggression	1089	57.77	829	43.98	1.75	[1.54, 1.99]	<0.001	***

## Data Availability

Restrictions apply to the availability of these data as they were obtained from and remain the property of the NCHA. Requests to access the datasets should be directed to the NCHA. Pre-processing and analysis scripts can be viewed on Github here: https://github.com/rebecca-murphy-ed/NCHA-analysis, accessed on 20 January 2026.

## Supplementary Materials

The following supporting information can be downloaded at https://www.mdpi.com/article/10.3390/nu18030458/s1. Table S1: Examples of write-in responses that were included and not included as GD when participants chose an “Other” Gender Identity; Supplementary Results: Table S2: Parameters of a quasi-Poisson regression model to predict the number of MH comorbidities of GD and non-GD individuals with an ED diagnosis. Model fitting was performed using the Complete dataset without PSM. Table S3: Parameters of a Quasipoisson regression model to predict the number of MH comorbidities of GD individuals with and without an ED diagnosis, using the Complete dataset without PSM. Table S4: Parameters of a Quasipoisson regression model to predict the number of MH Comorbidities of GD and Non-GD individuals with an ED Diagnosis. Model fitting was performed on a demographically matched sample from the full analytical sample with missing data values inferred using Multiple Imputation. Table S5: Parameters of a Quasipoisson regression model to predict the number of MH Comorbidities of GD and Non-GD individuals with an ED Diagnosis. Model fitting was performed on a demographically matched sample following imputation of missing values in the complete dataset. Refs. [84,85,86,87] are cited in Supplementary Materials file.

## Abbreviations

The following abbreviations are used in this manuscript:

ED	Eating Disorder
GD	Gender-Diverse
MH	Mental Health
PSM	Propensity Score Matching
