# Supplementary material for: Eating Disorders in People Who Identify as Gender-Diverse: Associations Between Gender Diversity, Eating Disorder Diagnosis, Minority Stress Experiences and Mental Health Comorbidity
Source: Nutrients. 2026 Jan 30;18(3):458. doi: 10.3390/nu18030458 (PMC12899334; doi:10.3390/nu18030458)
Supplement: Supplementary file 1 [file nutrients-18-00458-s001.zip › nutrients-4029744-supplementary.pdf]

Supplementary Information for Eating Disorders in People who Identify as Gender Diverse: What Are the Associations Between Gender Diversity, Eating Disorder Diagnosis, Minority Stress Experiences and Mental Health Comorbidities

November 2025

1 Supplementary Methods

Determining Gender Diverse Status

GD status was derived from the questions "What sex were you assigned at birth?", "Do you identify as transgender?" and "Which term do you use to describe your gender identity?" Diverse gender identities include trans man / woman, non-binary, agender, genderqueer and genderfluid. Participants were classed as GD if they endorsed a diverse gender identity from the suggested list of terms or from a write-in option; if they identified as transgender; or if they chose a gender identity that did not align with their birth sex. Write-in options were screened and participants giving ironic or offensive identities were not counted as GD. Similarly, participants who selected "other" and used the write-in box to state that they did not endorse the concept of gender identity were not counted as GD. Examples of screened write-in options with the screening decision are given in Table S1. Participants were not included in the analytic sample if they did not answer any of these questions in a manner that allowed them to be identified as gender diverse or not.

Table S1: Examples of write-in responses that were included and not included as GD when participants chose an "Other" Gender Identity.

GD	Aeroace
	Fluid
	Neutrois
	Pangender
	Queer
	She/They
	They/He
	Two Spirit
Not GD	Apache Attack Helicopter
	Bisexual
	Don't care
	Don't know
	Gender is a social construct
	Human
	None
	This is a stupid question

Multiple Imputation of Missing Data

Imputed variables comprised number of MH conditions, birth sex, age, gender identity, ethnicity and parent education level. Age and MH conditions were imputed using predictive mean matching; gender identity, parent education level and ethnicity were imputed using polytomous logistic regression; birth sex was imputed using logistic regression. MI was performed using the mice package (version 3.18.0) [1] in R version 4.5.1 [2]. Variables were imputed based on birth sex, age, gender identity, ethnicity and parent education level. We created five imputed datasets, using 20 sampling iterations per variable for each imputed dataset. MI was performed separately for the GD+ED group with the ED group and the GD+ED group with the GD group.

Demographic Matching and Model Fitting using Multiply Imputed Datasets

Following MI, downstream analyses were performed using the MatchThem package [3] (version 1.2.1) in R. Covariate matching using Nearest Neighbour Propensity score matching was performed within each dataset. Following matching, we used a quasi-Poisson regression model as the predictor for each imputed dataset, to infer the relationship between analytic group (GD+ED vs ED groups and GD+ED vs GD group) and number of comorbid mental health conditions. Demographic variables were included in the model as covariates. Parameter estimates were then pooled across the datasets using Rubin's rules [4].

Supplementary Results

Sensitivity Analysis: Impact of PSM - Analysis Using Complete Dataset

We used PSM to minimize demographic differences between the GD+ED and comparator groups. However, as one-to-one matching was used, this resulted in a large number of cases being excluded from downstream analysis. To confirm that this did not bias our findings, we repeated the regression analysis using the Complete dataset.

Table S2 shows parameter estimates of a quasipoisson regression model to predict the number of MH comorbidities of GD and non-GD individuals with an ED diagnosis, using the Complete dataset prior to PSM. Using the Complete dataset, we found that for individuals with an ED, being GD was associated with a significantly higher number of additional MH diagnoses, with a model parameter very similar to the analysis using the Matched ED group.

Table S2: Parameters of a quasi-Poisson regression model to predict the number of MH comorbidities of GD and non-GD individuals with an ED diagnosis. Model fitting was performed using the Complete dataset without PSM. Abbreviations: PEL - Parent Education Level.

Parameter	Coefficient	Standard Error	z value	p	
Intercept	0.88	0.022	39.83	$< 2e^{-16}$	***
Is GD	0.41	0.015	28.18	$< 2e^{-16}$	***
Age-Years	0.0063	0.00087	7.21	$5.61e^{-13}$	***
Birth Sex-Male	0.072	0.019	3.72	0.00020	***
PEL-Postgraduate	-0.052	0.013	-4.15	$3.32e^{-05}$	***
PEL-No College	0.090	0.014	6.31	$2.88e^{-10}$	***
Ethnicity-Multiracial	0.064	0.016	4.13	$3.70e^{-05}$	***
Ethnicity-Hispanic	-0.15	0.022	-6.69	$2.23e^{-11}$	***
Ethnicity-Asian	-0.17	0.025	-6.81	$9.70e^{-12}$	***
Ethnicity-Black	0.0034	0.0038	0.089	0.93	
Ethnicity-Other	0.057	0.037	1.56	0.12	

Table S3 shows parameter estimates of a quasipoisson regression model to predict the number of MH comorbidities of GD individuals with and without an ED diagnosis, using the Complete dataset prior to PSM. Using the Complete dataset, we found that for GD individuals and ED diagnosis was associated with a significantly higher number of additional MH diagnoses, with a model parameter very similar to the analysis using the Matched GD group.

Table S3: Parameters of a Quasipoisson regression model to predict the number of MH comorbidities of GD individuals with and without an ED diagnosis, using the Complete dataset without PSM. Abbreviations: PEL - Parent Education Level.

Parameter	Coefficient	Standard Error	z value	p	
Intercept	1.46	0.068	21.48	$< 2e^{-16}$	***
Has ED	1.30	0.046	28.37	$< 2e^{-16}$	***
Age-Years	0.028	0.0028	10.00	$< 2e^{-16}$	***
Birth Sex-Male	-0.51	0.037	-13.79	$< 2e^{-16}$	***
PEL-Postgraduate	-0.024	0.035	-71	0.48	
PEL-No College	0.092	0.040	2.29	0.022	*
Ethnicity-Multiracial	-0.0024	0.043	-0.056	0.96	
Ethnicity-Hispanic	-0.64	0.061	-10.43	$< 2e^{-16}$	***
Ethnicity-Asian	-0.92	0.052	-6-17.65	$< 2e^{-16}$	***
Ethnicity-Black	-0.48	0.083	-5.76	$8.22e^{-09}$	***
Ethnicity-Other	-0.26	0.10	-2.52	0.012	*

Sensitivity Analysis: Impact of Missing Data on Differences in MH Comorbidities and demographic characteristics of GD and Non-GD individuals with an ED Diagnosis

As PSM requires a complete dataset, we initially excluded participants with any missing data. However, as missingness is rarely comppletely at random across a dataset [5], analysis on only complete cases may bias findings. To mitigate this, we used Multiple Imputation via Chained Equations to infer plausible missing values for participants with missing demographic data. Five multiply imputed datasets were created. PSM was completed to demographically match the ED group to the GD+ED group within each imputed dataset; the regression analysis was completed; and the resulting model coefficients were pooled. Table S4 shows the pooled parameter estimates, showing that GD identity remains significantly associated with additional MH diagnoses for individuals with a diagnosed ED and that this finding is not primarily an artifact of missing data.

Table S4: Parameters of a Quasipoisson regression model to predict the number of MH Comorbidities of GD and Non-GD individuals with an ED Diagnosis. Model fitting was performed on a demographically matched sample from the full analytical sample with missing data values inferred using Multiple Imputation. Abbreviations: PEL - Parent Education Level. Note that categorical variables are dichotomised against the modal category.

Parameter	Coefficient	Standard Error	z value	p	
Intercept	0.89	0.051	17.47	$< 2e^{-16}$	***
Is GD	0.38	0.020	19.25	$< 2e^{-16}$	***
Age-Years	0.0080	0.0021	3.73	$2.13e^{-04}$	***
Birth Sex-Male	0.096	0.039	2.48	0.039	**
PEL-Postgraduate	-0.054	0.022	-2.47	0.014	**
PEL-No College	0.081	0.026	3.16	$1.61e^{-03}$	***
Ethnicity-Multiracial	0.057	0.025	2.32	0.021	***
Ethnicity-Hispanic	-0.19	0.055	-3.42	$7.06e^{-04}$	***
Ethnicity-Asian	-0.089	0.060	-1.48	-0.14	
Ethnicity-Black	0.12	0.086	1.45	0.59	
Ethnicity-Other	0.015	0.079	0.19	0.85	

Sensitivity Analysis: Impact of Missing Data on Differences in MH Comorbidities and demographic characteristics of GD individuals with and without an ED Diagnosis: Analysis Using Multiply Imputed Dataset

To ensure that our findings were not influenced by the exclusion of participants with missing data, we created five datasets with plausible missing values inferred using Multiple Imputation. Following PSM, we completed a similar Quasipoisson regression within each imputed dataset and the resulting model coefficients were pooled. Table S5 shows the pooled parameter estimates, showing that ED diagnosis remains significantly associated with additional MH diagnoses for GD individuals.

Table S5: Parameters of a Quasipoisson regression model to predict the number of MH Comorbidities of GD and Non-GD individuals with an ED Diagnosis. Model fitting was performed on a demographically matched sample following imputation of missing values in the complete dataset. Abbreviations: PEL - Parent Education Level. Note that categorical variables are dichotomised against the modal category Abbreviations: PEL - Parent Education Level. Note that categorical variables are dichotomised against the modal category.

Parameter	Coefficient	Standard Error	t	p	
Intercept	0.49	0.059	8.28	$6.85e^{-15}$	***
Has ED	0.79	0.026	30.34	$< 2e^{-16}$	***
Age-Years	0.0091	0.0024	3.73	$2.58e^{-04}$	***
Birth Sex-Male	-0.048	0.040	-1.20	0.85	
PEL-Postgraduate	-0.054	0.024	-2.28	0.30	
PEL-No College	0.081	0.026	3.16	$1.61e^{-03}$	***
Ethnicity-Multiracial	0.058	0.027	2.12	0.035	*
Ethnicity-Hispanic	-0.22	0.053	-4.11	$4.11e^{-05}$	***
Ethnicity-Asian	-0.10	0.061	-1.67	-0.095	
Ethnicity-Black	0.014	0.086	0.16	0.87	
Ethnicity-Other	-0.033	0.079	-0.42	0.68	

References

[1] Stef Van Buuren and Karin Groothuis-Oudshoorn. mice: Multivariate imputation by chained equations in r. *Journal of Statistical Software*, 45(3):1–67, 2011.

[2] R Core Team. R: A language and environment for statistical computing, 2020.

[3] Farhad Pishgar, Noah Greifer, Clémence Leyrat, and Elizabeth Stuart. MatchThem: matching and weighting after multiple imputation. *arXiv preprint arXiv:2009.11772*, 2020.

[4] Roderick JA Little and Donald B Rubin. *Statistical analysis with missing data.* John Wiley & Sons, Hoboken, New Jersey, 2019.

[5] James R Carpenter, Michael G Kenward, and Ian R White. Sensitivity analysis after multiple imputation under missing at random: A weighting approach. *Statistical Methods in Medical Research*, 16(3):259–275, 2007.
